# Assessment of Freeze-Dried Immobilized *Lactobacillus casei* as Probiotic Adjunct Culture in Yogurts

**DOI:** 10.3390/foods8090374

**Published:** 2019-09-01

**Authors:** Dimitra Dimitrellou, Panagiotis Kandylis, Yiannis Kourkoutas

**Affiliations:** 1 Department of Molecular Biology and Genetics, Democritus University of Thrace, Alexandroupolis 68100, Greece; 2 Department of Chemistry, University of Patras, Patras 26504, Greece; 3 Department of Food Science and Technology, School of Agriculture, Aristotle University of Thessaloniki, P.O. Box 235, Thessaloniki 54124, Greece

**Keywords:** probiotic, *Lactobacillus casei*, freeze drying, volatiles, multiplex PCR, GC-MS, apple pieces, casein

## Abstract

Freeze-dried immobilized *Lactobacillus casei* ATCC 393 on casein and apple pieces were assessed as a probiotic adjunct culture for novel probiotic yogurt production. The effect of probiotic culture on physicochemical characteristics, probiotic cell survival, volatile aroma compounds, and sensory quality were evaluated during 28 days of storage at 4 °C. The use of *L. casei* resulted in lower pH values (3.92–4.12), higher acidity (0.88–1.10 g lactic acid/100 g of yogurt), and lower syneresis (40.8%–42.6%) compared to traditionally produced yogurt (pH 4.29; acidity 0.83 g lactic acid/100 g of yogurt; syneresis 44.1%). Microbiological and strain-specific multiplex PCR (Polymerase Chain Reaction) analysis confirmed that immobilized *L. casei* ATCC 393 cells were detected in yogurts at levels >7 log cfu g^−1^ after 28 days. In addition, probiotic supplementation significantly affected the concentrations of key volatile compounds, like acetic and other organic acids, 2-ethyl-1-hexanol, acetoin, and 2-butanone, as revealed by GC-MS (Gas Chromatography–Mass Spectrometry) analysis. Finally, the sensory evaluation demonstrated that the new products exhibited improved characteristics compared to traditionally produced yogurts.

## 1. Introduction

Yogurt is a fermented dairy product that is widely consumed throughout the world and with thousands of years of history [1]. In this vein, there is nowadays a growing interest in developing novel dairy products containing probiotic microorganisms, such as bifidobacteria and lactic acid bacteria, with a great potential for promoting human health [2,3]. Indeed, yogurt has been characterized as an ideal carrier for the delivery of probiotics and numerous studies, describing the development of novel probiotic products, are expected within the next years, leading to increased consumer acceptability, as well as profit-making for manufacturers [4]. However, the most important issue in probiotic yogurt development is to ensure cell viability until the time of consumption in sufficient numbers, in order to exert their numerous health benefits, a fact that is not usually fulfilled [5].

*Lactobacillus casei* ATCC 393 is a strain with many applications in food production [6,7,8] and several probiotic properties, such as survival under in vitro gastrointestinal conditions [9] and survival during the passage through the gastrointestinal tract in a rat model, potential regulation of intestinal microbiota [10], as well as tumor-inhibitory, anti-proliferative, and pro-apoptotic effects [11,12].

Immobilization is a method that has been used to increase the viability of added cultures during food production and storage [9,13]. Among others, milk proteins, such as caseins, have already been used as probiotic cells carrier in dairy products [14,15,16]. The addition of fruits, like apples, is a common practice in yogurt production due to the numerous health benefits associated with their consumption. Apples were selected in the present study since they are an excellent source of several phenolic compounds and dietary fibers, and also possess high total antioxidant capacity [17,18]. Dietary fibers have been characterized as functional ingredients owe to their prebiotic activity, and in combination with probiotics, may affect positively gastrointestinal functions [19,20]. Such characteristics make apple pieces a suitable delivery vehicle for probiotics either used as novel probiotic dehydrated fruits [21] or incorporated in dairy products when bearing immobilized probiotic cells [10,22].

Hence, the aim of the present study was to evaluate the use of freeze-dried immobilized *Lactobacillus casei* ATCC 393 on casein and apple pieces as a probiotic adjunct culture for novel probiotic yogurt production. Freeze-dried immobilized *L. casei* cells were used in order to comply with industrial and commercial needs. In addition, in order to evaluate successful yogurt production, the main physicochemical, microbiological, and aroma characteristics of novel yogurts were evaluated. Finally, a sensory evaluation was conducted to assess the acceptance of the new yogurt by consumers.

## 2. Materials and Methods 

### 2.1. Strains

*Lactobacillus casei* ATCC 393 (DSMZ, Braunschweig, Germany) was used as a probiotic type strain. The thermophilic starter, CH-1 (Chr. Hansen, Hørsholm, Denmark) was used as commercial yogurt starter culture. Both were prepared for yogurt production as described in a previous study [9]. In brief, CH-1 culture was activated by adding a 50 U sachet to 500 mL of sterile 140 g L^−1^ skim milk, while *L. casei* ATCC 393 was grown in de Man, Rogosa and Sharpe (MRS) broth at 37 °C.

### 2.2. Immobilization of L. casei

Cell immobilization on apple pieces and casein was carried out as described in a previous study [13]. In brief, apples were cut in small pieces, placed in a conical flask containing *L. casei* cells in MRS broth and were left undistributed at 37 °C for 24 h. When immobilization was completed, the fermented liquid was decanted, and the immobilized cells were washed twice with sterilized 1/4 Ringer’s solution. For cell immobilization on casein, *L. casei* culture was added to commercial pasteurized skim bovine milk that was heated to 37 °C and commercial rennet was then added. After curd formation and cloth filtration at room temperature, which was carried out overnight, the resulting curd was cut in small cubes.

### 2.3. Freeze Drying of Free and Immobilized L. casei Cells

Freeze drying of free and immobilized *L. casei* cells was carried out according to a previous study, using deproteinized whey as lyoprotectant in the case of free cells, and lactose and monosodium glutamate in the case of immobilized cells on apple pieces and casein, respectively [13]. 

### 2.4. Yogurt Production

A commercial pasteurized bovine milk (“Small Family Farms”, Delta S.A., Greece, 37 g L^−1^ fat, 47 g L^−1^ sugars, 33 g L^−1^ protein) was tempered at 42–45 °C and the freeze-dried *L. casei* was added either in free (0.3 g in 150 mL milk) or immobilized form (1.9 g in 150 mL milk). Prior to milk addition, the freeze-dried cultures were rehydrated in 5 mL milk for 15 min. After 30 min, all milk samples were inoculated with the activated CH-1 culture (0.3% (*v*/*v*)). Milk samples were fermented until pH 4.6. Thereafter, the yogurts were stored at 4 °C for 28 days. Yogurt with only CH-1 culture was also produced (control sample) in order to allow comparison of results. Therefore, four different yogurts were produced: yogurt with CH-1 culture (C); yogurt with freeze-dried free *L. casei* cells and CH-1 culture (FLC); yogurt with freeze-dried immobilized *L. casei* cells on apple pieces and CH-1 culture (ILCA); yogurt with freeze-dried immobilized *L. casei* cells on casein and CH-1 culture (ILCC).

### 2.5. Determination of Cell Viability

#### 2.5.1. Determination of *S. thermophilus*, *L. bulgaricus*, and *L. casei* Viability

The viability of *Streptococcus thermophilus*, *Lactobacillus delbrueckii* subsp. *bulgaricus*, and *L. casei* was measured using yogurt samples of 10 g and following the procedure described in a previous study [9]. 

#### 2.5.2. Determination of *L. casei* ATCC 393 Levels

To determine the levels of *L. casei* ATCC 393 strain in probiotic yogurt samples, a methodology recently described by Sidira et al. [10] was followed, based on a multiplex PCR assay [23].

### 2.6. Physicochemical Analysis

#### 2.6.1. pH and Titratable Acidity of Yogurts

Yogurts pH was measured using a pH meter and titratable acidity was determined by titration using 0.1 mol L^−1^ NaOH and phenolphthalein as an indicator, and expressed as g of lactic acid per 100 g yogurt [9].

#### 2.6.2. Susceptibility to Syneresis and Water Holding Capacity

The yogurt “susceptibility to syneresis” (STS) was determined using 50 mL of unstirred yogurt spread evenly on a Whatman No.1 filter paper (Whatman Ltd., Maidstone, England) in a funnel at 4 °C. After 5 h of drainage, the volume of whey collected in a beaker was measured, multiplied by 2 and expressed as STS (%) [3].

The water holding capacity (WHC) was determined by centrifugation of a 10 g yogurt sample at 4500 rpm for 30 min at 10 °C, and calculated using the following equation:

WHC (%) = (1 − W_1_/W_2_) × 100, (1)

where W_1_ = weight of whey after centrifugation, W_2_ = yogurt sample weight [3].

### 2.7. Solid-Phase Microextraction GC-MS Analysis

Yogurts samples after 14 days of refrigerated storage were used to study the volatile composition by solid-phase microextraction (SPME) GC-MS analysis. The procedure described in a previous study [14] was followed, using 7 g of yogurt. Each determination was carried out in triplicate, and the mean data are presented.

### 2.8. Sensory Evaluation of Yogurts

The yogurts were tested for their sensory characteristics as described in a previous study [9]. More specifically, the evaluation was conducted by 20 untrained panelists (10 females and 10 males aged 20–45 years) familiar with the consumption of yogurts. All samples were presented in uniform plastic cups at 15 °C, coded with random three-digit numbers. Water was provided between samples to cleanse the palate. The yogurts were evaluated for color, sweet odor, sourness, smoothness, sweetness, viscosity, and overall acceptability using a 9-point hedonic scale ranging from 1 (“dislike extremely”) to 9 (“like extremely”).

### 2.9. Statistical Analysis

All experiments were carried out in triplicate and duplicate samples were collected for each analysis. Significance was established at *p* < 0.05. The results were analyzed for statistical significance with ANOVA, and Tukey’s honest significant difference (HSD) test was used to determine significant differences among results; coefficients, ANOVA tables, and significance (*p* < 0.05) were computed using Statistica version 5.0 (StatSoft Inc., Tulsa, OK, USA). 

## 3. Results and Discussion

### 3.1. Production of Novel Probiotic Yogurts with Immobilized L. casei ATCC 393

Yogurt is a traditional dairy product obtained after lactic acid fermentation, usually of two microorganisms, namely *Streptococcus thermophilus* and *Lactobacillus delbrueckii* subsp. *bulgaricus,* with growing worldwide production and consumption. However, research on the development of novel yogurt-based products is increased in recent years in order to fulfill two main demands of consumers. Firstly, the request for more healthy foods has led to the development of novel functional dairy products with potential health benefits like probiotic yogurts [24] and, secondly, the increasing popularity of yogurts with new flavors has resulted in the development of novel yogurts with different sources of fruit flavorings [25,26]. In this study, both requirements were fulfilled by using immobilized *L. casei* ATCC 393 cells on apple pieces or casein. *Lactobacillus casei* ATCC 393 was mainly selected due to its satisfying technological and health-promoting characteristics, but also due to its capability to survive the harsh conditions of the gastrointestinal tract, as was demonstrated both in vitro, in simulated gastrointestinal conditions [9], and in vivo, in a Wistar rat model [10]. Both apple and casein have been used as immobilization supports in several foods, and have also proven capable of protecting *L. casei* cells during freeze drying [13].

### 3.2. Physicochemical Characteristics of Novel Probiotic Yogurts 

**Figure 1:**
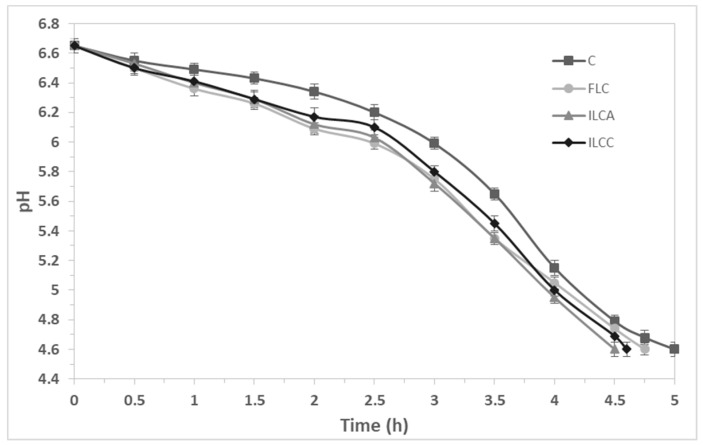
Acidification kinetics during yogurt production. (C = Yogurt produced with CH-1 culture; FLC = yogurt produced with freeze-dried free *L. casei* ATCC 393 cells and CH-1 culture; ILCA = yogurt produced with freeze-dried immobilized *L. casei* ATCC 393 cells on apple pieces and CH-1 culture; ILCC = yogurt produced with freeze-dried immobilized *L. casei* ATCC 393 cells on casein and CH-1 culture).

The acidification trend in yogurt fermentation is presented in Figure 1. The use of different cultures had no significant effect on milk acidification. The small observed differences were not important from a technological point of view. The maximum rate of pH decline occurred between 2.5 and 4.5 h after milk inoculation, which is in accordance with previous studies using the same starter culture and different probiotics [24]. The total acidification time was 5, 4.75, 4.6, and 4.5 h for C, FLC, ILCC, and ILCA yogurts, respectively, showing a slight reduction with the use of *L. casei*, as was also reported in previous studies [24,27]. 

As expected, a postacidification effect was observed in yogurts resulting in lower pH values during storage (Table 1). No significant differences were observed in the pH values at the 1st day and until 7th day of storage (ranging between 4.50 and 4.55 at 1st day and 4.37 and 4.46 at 7th day). However, after the 14th day, the use of *L. casei* significantly affected (*p* < 0.05) the pH values, and as the storage continued, this effect became more significant (*p* = 0.028, *p* = 0.042, and *p* = 0.003 for 14th, 21st, and 28th day of storage respectively). The use of *L. casei*, especially in immobilized form, led to lower pH values compared to the control yogurt, which is in accordance with previous studies [24]. At the end of storage, the pH values were 4.29 for C, 4.12 for FLC, 4.04 for ILCA, and 3.92 for ILCC samples. The same effect was also observed in fermented milks using microencapsulated *L. casei* ATCC 393 cells [9]. Similar pH values (3.9 to 4.3) were also reported in yogurts with several probiotic cultures [5,7,28]. 

Another way to study postacidification of yogurts is to determine their acidity. The effect of storage time on yogurts acidity is presented in Table 1. Initial values varied from 0.72% to 0.76% and no significant differences were observed between the 1st and the 7th day of storage. However, after the 14th day of storage, the use of *L. casei*, especially in immobilized form, significantly affected (*p* < 0.05) yogurt acidity (*p* = 0.025, *p* = 0.003, and *p* = 0.002 for 14th, 21st, and 28th day of storage, respectively), leading to higher acidities compared to the control yogurt, as reported in previous studies [9,24]. At the end of the storage period, the acidities were 0.83 for C, 0.88 for FLC, 1.04 for ILCA, and 1.10 for ILCC and similar values were previously reported [6]. Since postacidification depends on the microorganisms used and their viable counts during storage, these results may be attributed to the high viability of *L. casei* due to the protection by immobilization and the acid-resistant nature of the strain [9,29], as well as its capability to produce higher amounts of lactic acid if compared to other probiotics [24]. 

Whey separation is a common defect in fermented milk products [30], which affects consumer perceptions. In the present study, STS values were significantly affected (*p* < 0.05) by the use of *L. casei* and especially in immobilized form, leading to lower STS values. This may be attributed to the high WHC of casein and apple pieces. Milk proteins, like casein, are reported to reduce the STS values [3], while pectin, contained in apple pieces, acts as a stabilizer of yogurt gel matrix and also reduces STS values. Storage affected only the yogurts produced with *L. casei* cells and significantly higher values of syneresis were observed at the end of storage, which may be attributed to the significantly higher values in acidity. Increased syneresis with storage time is usually associated with severe casein network rearrangements [31]. A similar trend was observed in the values of WHC. More specifically, after 28 days of storage, WHC values were 53.6%, 55.5%, 57.9%, and 57.7%, for C, FLC, ILCA, and ILCC yogurts, respectively.

### 3.3. Microbiological Analysis of Novel Probiotic Yogurts 

**Figure 2:**
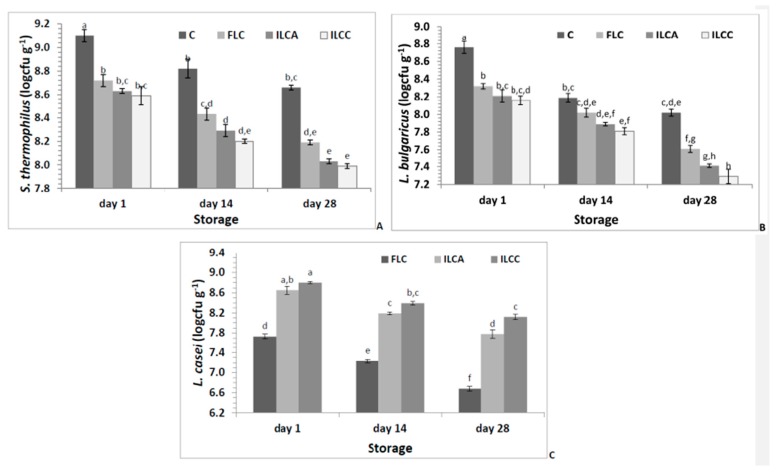
Viability of microorganisms used in yogurt production during refrigerated storage; (**A**) *Streptococcus thermophilus*, (**B**) *Lactobacillus delbrueckii* subsp. *bulgaricus*, (**C**) *Lactobacillus casei* ATCC 393. (C = Yogurt produced with CH-1 culture; FLC = yogurt produced with freeze-dried free *L. casei* ATCC 393 cells and CH-1 culture; ILCA = yogurt produced with freeze-dried immobilized *L. casei* ATCC 393 cells on apple pieces and CH-1 culture; ILCC = yogurt produced with freeze-dried immobilized *L. casei* ATCC 393 cells on casein and CH-1 culture. Different letters in the columns indicate significant differences (*p* < 0.05)).

The numbers of viable cells of starter and adjunct cultures during 28 days of storage of the probiotic yogurts are presented in Figure 2. In some cases, the yogurt starter cultures produce inhibitory substances (such as hydrogen peroxide, bacteriocins, or lactic acid), which could potentially reduce the viability of probiotic bacteria [32]. Therefore, in the present study, the cultures were separately inoculated into milk. Firstly, milk was inoculated by *L. casei* cells, in order to provide the appropriate time to increase their numbers, and the commercial yogurt starters were then added after 30 min.

In all cases, even after storage for 28 days, the sum of yogurt starter bacteria (Figure 2A,B), *S. thermophilus* and *L. delbrueckii* ssp. *bulgaricus*, was above the minimum requirement of 10^7^ viable microorganisms per gram established by FAO/WHO [33]. *Streptococcus thermophilus*, in general, survives well (> 10^8^ cfu/mL) in fermented milk products during refrigerated storage, and this was also observed in all yogurt samples (Figure 2A). The addition of probiotic *L. casei* and the storage significantly affected (*p* < 0.05) the viability of *S. thermophilus* and *L. bulgaricus*, leading to reduced numbers. It is well known that the incorporation of probiotics into yogurts, together with starter cultures, leads to interactions between the growth patterns of each strain [24]. In all cases, the numbers of yogurt starters were significantly lower in yogurts with *L. casei* (FLC, ILCA, and ILCC) compared to the control sample (C), which may be attributed to their reduced stability due to the competitive action of probiotic *L. casei* [24,27]. *S. thermophilus* numbers were higher than those of *L. bulgaricus*, indicating better adaption of *S. thermophilus* cells to the presence of *L. casei.* A reduction in the viability of *L. bulgaricus* up to 30%–50% in the presence of *L. casei* has been also reported in previous studies [5,28], and may be attributed to the secretion of inhibitory metabolites produced by probiotics that may affect species of the same genus [5]. Indeed, in a previous study, four probiotic strains, namely *L. casei*, *L. acidophilus*, *L. plantarum*, and *L. rhamnosus*, led to a reduction of *S. thermophilus* and *L. bulgaricus* viability in yogurt, with *L. casei* presenting the most significant effect [24]. Apart from the antagonistic activity of *L. casei* towards the typical yogurt microflora and the marked acidification of the new products, no significant alterations were observed compared to the control samples, according to the preliminary sensory evaluation, as described below in detail.

**Figure 3:**
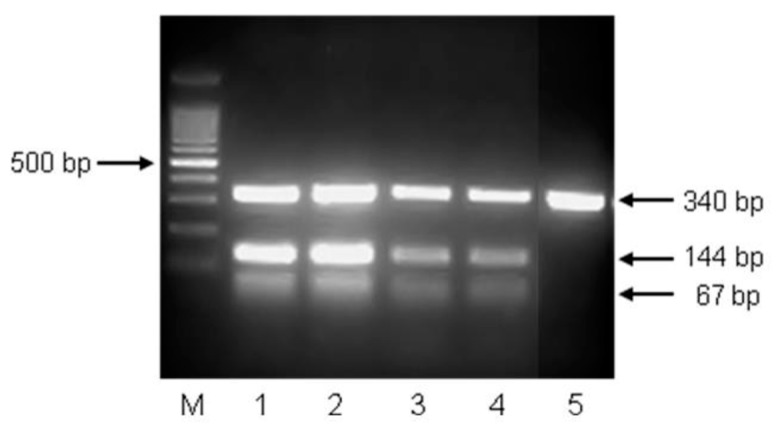
Molecular identification of *L. casei* ATCC 393 in yogurts after 28 days of refrigerated storage. Lane 1: pure culture of *L. casei* ATCC 393; Lane 2: probiotic yogurt with immobilized *L. casei* ATCC 393 cells on casein; Lane 3: probiotic yogurt with immobilized *L. casei* ATCC 393 cells on apple pieces; Lane 4: probiotic yogurt with free *L. casei* ATCC 393 cells; Lane 5: yogurt without *L. casei* ATCC 393 cells. PCR products of 67 and 144 bp are unique for *L. casei* ATCC 393, whereas the PCR product of 340 bp is universal for lactobacilli.

A critical issue in the development of probiotic fermented dairy products is to ensure the viability of probiotics during fermentation and storage. Figure 2C presents changes in *L. casei* ATCC 393 counts during refrigerated storage of probiotic yogurts. An increase in *L. casei* cells was observed during the fermentation process, resulting in 7.73, 8.65, and 8.80 log cfu/g at the first day of storage in FLC, ILCA, and ILCC, respectively. This significant growth of *L. casei* was also observed in previous studies [24] and is attributed to its capability to grow well in milk environment [34]. Immobilization significantly affected (*p* < 0.05) the viability of *L. casei* cells throughout storage and may be attributed to the protective effect of apple and casein [13]. More specifically, during storage, the numbers of immobilized cells were 0.9–1.0 log cfu g^−1^ higher than those of free cells. The duration of refrigerated storage significantly affected (*p* < 0.05) the viability of *L. casei*, resulting in a significant decline (1.05, 0.87, and 0.68 log cfu g^−1^ in FLC, ILCA, and ILCC, respectively). Similar results have been reported in previous studies with yogurts [5,24,27]. The numbers of *L. casei* cells in all probiotic yogurts during production and refrigerated storage (even after 28 days) were above the requirement of 6 log cfu g^−1^ for microorganisms (other than the starter culture) added to yogurt [35], and for probiotic food, according to US FDA and the food industry [36]. Of note, the use of immobilized cells led to even higher viabilities of *L. casei* cells, up to 7.78 and 8.12 log cfu g^−1^ for ILCA and ILCC, respectively. These high numbers also fulfill the demand for a daily intake of 10^8^–10^9^ probiotic microorganisms, in order to accomplish a probiotic action in the host [37], which may be easily achieved with the consumption of 10–100 g of novel yogurts. Furthermore, the high numbers of *L. casei* cells after 28 days of storage were also corroborated by multiplex PCR assay (Figure 3), which provides accurate, reliable, convenient, and sensitive detection of *L. casei* ATCC 393 cells compared to traditional methods [2].

### 3.4. Aroma-Related Compounds of Novel Probiotic Yogurts 

Probiotic yogurts, containing either free (FLC) or immobilized *L. casei* cells (ILCA and ILCC), and yogurt produced with only the commercial starter (C) were analyzed for their aroma-related compounds (SPME GC-MS technique). From a qualitative point of view, no significant differences were observed in the numbers of compounds between yogurt samples, confirming the findings of previous studies reporting that probiotics, when used in combination with traditional yogurt starters, do not influence the formation of major aroma compounds, but rather their concentrations [38]. The compounds identified in the yogurts are listed by chemical group in Table 2.

#### 3.4.1. Esters

Only 4 esters were detected belonging to the group of ethyl esters. Ethyl esters originated from the enzymatic or chemical esterification of acids with ethanol [39] and are typically characterized by a fruity aroma [40]. All of them were detected in low concentrations, since the presence of esters in yogurts is associated with extended storage [39].

#### 3.4.2. Organic Acids

Organic acids presented the second highest concentration after ketones. Acetic acid is an important compound in yogurts and was found in concentrations similar to other studies [41]. Other acids that were detected and are frequently found in dairy products were butanoic, hexanoic, octanoic, nonanoic, and decanoic acids [16,24,26,40]. The use of *L. casei* resulted in significantly (*p* < 0.05) higher concentrations of butanoic, hexanoic, and octanoic acids, while immobilized cells resulted in significantly higher concentrations of acetic and decanoic acids. Previous studies have correlated the activity of *L. casei* with the formation of organic acids in yogurts [24], fermented milks [34], and cheeses [42]. 

#### 3.4.3. Alcohols

Apart from ethanol, which was detected in all yogurts, 10 other alcohols were detected in total (Table 2). 3-Methyl-1-butanol, having an alcoholic, floral note, was detected in FLC and ILCA samples, while 1-hexanol, with fruity notes, was present in C and ILCC. Secondary alcohols like 2-heptanol (only in ILCA yogurt) and 2-nonanol (present in all samples) were also detected and are usually formed by the enzymatic reduction of the corresponding methyl ketones, which themselves originate from fatty acids by β-oxidation or from β-ketoacids [14]. These alcohols are usually identified in several dairy products, including fermented milk [41], yogurt [24] and cheeses [14]. 1-Octen-3-ol, present only in C and ILCA samples, is well known for its raw mushroom odor [40]. Finally, 2-ethyl-1-hexanol, an important flavor compound in dairy products, was detected in all samples and its content was significantly (*p* < 0.05) affected by the use of *L. casei*, resulting in higher concentrations. It is an important flavor compound produced by *L. casei*, as was also found in previous studies with yogurts and cheeses [24,42].

#### 3.4.4. Aldehydes

Acetaldehyde is considered as one of the key volatile compounds for the flavor of yogurts, responsible for the characteristic green apple or nutty flavor [39], and was among the detected aldehydes. It is derived from the cleavage of threonine, with the action of a threonine aldolase to form acetaldehyde and glycine [40]. This enzyme occurs in both *S. thermophilus* and *L. delbrueckii* subsp. *bulgaricus*, but that derived from *L. bulgaricus* acts better at 42 °C and therefore in yogurts, acetaldehyde is mainly produced by *L. delbrueckii* subsp. *bulgaricus* [44]. No significant differences (*p* > 0.05) due to the activity of *L. casei* were observed, supporting the previously mentioned conclusion. In addition, previous studies showed that yogurt starter CH-1, is capable of producing acetaldehyde in considerable amounts [45]. Hexanal, detected in yogurts with *L. casei* (FLC, ILCA, and ILCC), and heptanal, detected in all samples, provide the green note of immature fruit, while octanal and nonanal, also detected in all samples, are described as having an aromatic note resembling orange [40]. The use of *L. casei* resulted in significantly (*p* < 0.05) higher concentrations of octanal and (*E,E*)-2,4-heptanedial, while the addition of immobilized cells significantly increased (*p* < 0.05) the concentrations of nonanal and (*E*)-2-nonenal.

#### 3.4.5. Ketones

Ketones were the group with the highest concentration in the yogurts of the present study. Acetone, detected in all samples with no significant (*p* > 0.05) differences, has a sweet, fruity aroma, and is known to influence the aroma and flavor of yogurt [39]. It originates usually from milk, but it is also produced by the action of yogurt bacteria, as reported previously, using the same yogurt starter CH-1 [45]. Several methyl ketones, with fruity, floral, and musty notes, were detected in all yogurts, such as 2-butanone, 2-pentanone, 2-heptanone, 2-octanone, 2-nonanone, 2-undecanone, and 2-tridecanone [40]. In addition, the concentrations of all ketones, apart from 2-nonanone, were significantly (*p* < 0.05) higher in yogurt samples containing either free or immobilized *L. casei*. These methyl ketones have been characterized as significant volatile organic compounds in samples fermented with *L. casei* and originate mainly from lipolysis, oxidation, and decarboxylation of fatty acids [24]. 3-Hydroxy-2-butanone (acetoin), with creamy and butter-like flavor, was present in all yogurt samples. It is known that commercial starter CH-1 produces acetoin in lower contents compared to other cultures [45], and the same result was observed in our case. However, *L. casei* resulted in significantly (*p* < 0.05) higher concentrations. Diacetyl (2,3-butanedione) was also detected in all yogurts and is responsible for a buttery flavor that is considered positive at elevated concentrations [39]. No significant differences were observed between the samples regarding the content of diacetyl, which in combination with the high levels of acetoin, is responsible for the mild, pleasant, buttery taste that is critical to the rich perception of yogurt [39].

#### 3.4.6. Lactones

Lactones are generally characterized by very pronounced fruity attributes; however, they are also recognized as important compounds for their ability to suppress the unpleasant flavor of other components, such as fatty acids, thus providing a milder and pleasant character [15]. Significantly (*p* < 0.05) higher concentrations of δ-dodecalactone were detected in yogurts with *L. casei* (FLC, ILCA, and ILCC). The correlation of *L. casei* with the production of this lactone was also reported in previous studies using *L. casei* in Feta-type and whey cheeses [16,42], confirming the positive effect of *L. casei* on the aroma of the final product.

#### 3.4.7. Total Volatile Compounds

No significant differences (*p* > 0.05) were observed in total concentration of esters and alcohols. However, immobilized *L. casei* cells (ILCA and ILCC) led to significantly (*p* < 0.05) higher concentrations of organic acids, aldehydes, ketones, and total compounds, compared to the C sample. These results may be attributed to the relatively high viable counts of *L. casei*, especially in immobilized form during the storage period, which resulted in high metabolic activities altering the volatile profile of yogurts. Therefore, it may be concluded that the addition of *L. casei* ATCC 393 significantly affected the formation of minor volatile compounds, which may also have an impact on the final organoleptic quality.

### 3.5. Preliminary Sensory Evaluation of Novel Probiotic Yogurts

**Figure 4:**
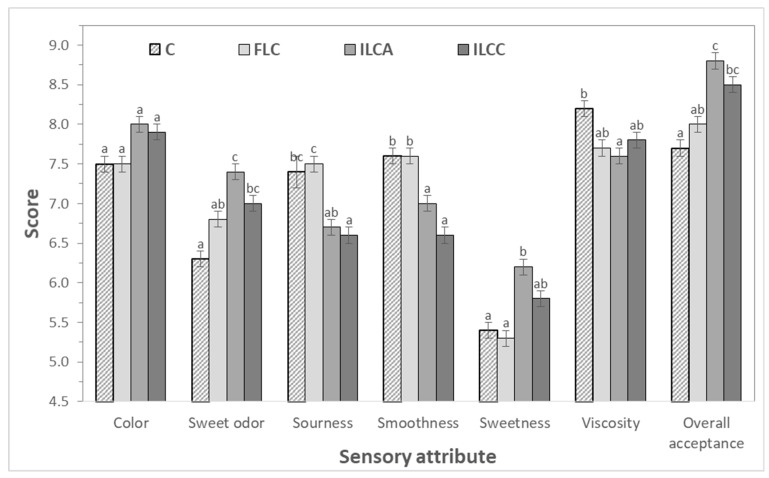
Sensory evaluation of novel probiotic yogurts produced using free (FLC) or immobilized *L. casei* ATCC 393 cells on apple pieces (ILCA) and casein (ILCC) in comparison to control samples (C). Different letters in the columns in the same sensory attribute indicate significant differences (*p* < 0.05).

The use of *L. casei* resulted in improved sensory characteristics (Figure 4), and significantly affected all the sensory attributes apart from color, where all yogurts received similar values. A slight, but not significant increase, was observed in color with the use of immobilized cells. 

A more significant effect was observed in smoothness (*p* = 0.005), with ILCA and ILCC receiving the lowest scores due to the presence of solid immobilized cells, and in sweet odor (*p* = 0.007), with ILCA and ILCC receiving the highest scores (7.4 and 7.0, respectively) followed by FLC (6.8) and C (6.3). Sourness was also significantly affected (*p* = 0.017) by the use of *L. casei* and ILCA and ILCC scored lower values due to the lower pH and higher acidities during storage. Less significant was the effect on viscosity (*p* = 0.046), but more significant (*p* = 0.005) on overall acceptability. According to the GC–MS analysis, yogurts with immobilized cells contained higher concentrations of volatiles like acetoin and 2-butanone that mostly contribute to the typical aroma and flavor of yogurt, in combination with acetaldehyde, diacetyl, and acetone [39]. This may explain the higher scores of the yogurts with immobilized cells during the sensory evaluation. In addition, more fruity notes were associated to ILCA sample due to the presence of apple pieces. This result is also explained by the higher concentrations of compounds like octanol, acetic acid, 2-butanone, and acetoin in ILCA that have been correlated with fruity flavor in yogurts and other dairy products [46,47]. Similarly, significant improvement of sensory characteristics was also previously recorded in dairy products containing *L. casei* as an adjunct or starter culture [5,9,16,42,48].

## 4. Conclusions

The present study clearly showed that the addition of free or immobilized *L. casei* on apple pieces and casein had a positive effect on yogurt characteristics. Their use led to novel probiotic products with improved quality, as confirmed by the sensory evaluation and the SPME GC-MS analysis of aroma-related compounds. Importantly, apple pieces and casein retained the viability of *L. casei* cells during refrigerated storage at the essential concentration for providing health benefits. These results are considered very promising for the design and development of novel dairy products that will combine the probiotic benefits with new or improved flavor, enhancing their acceptability by consumers.

## Figures and Tables

**Table 1:** Physicochemical characteristics of yogurts during storage at 4 °C for up to 28 days.

Analysis	Storage (Days)	Yogurt ^1^			
		C	FLC	ILCA	ILCC
**pH**	1	4.55 ± 0.05 ^a^	4.50 ± 0.03 ^a^	4.52 ± 0.05 ^a^	4.51 ± 0.02 ^a^
	7	4.46 ± 0.04 ^ab^	4.37 ± 0.01 ^b^	4.39 ± 0.02 ^ab^	4.42 ± 0.03 ^a^
	14	4.32 ± 0.02 ^b,XY^	4.35 ± 0.02 ^bc,X^	4.30 ± 0.03 ^bc,XY^	4.20 ± 0.01 ^b,Y^
	21	4.30 ± 0.03 ^b,X^	4.22 ± 0.02 ^cd,XY^	4.20 ± 0.01 ^cd,XY^	4.18 ± 0.01 ^b,Y^
	28	4.29 ± 0.02 ^b,X^	4.12 ± 0.04 ^d,Y^	4.04 ± 0.02 ^d,YZ^	3.92 ± 0.02 ^c,Z^
**Acidity** **(g lactic acid/100 g yogurt)**	1	0.75 ± 0.01	0.72 ± 0.02 ^b^	0.75 ± 0.03 ^b^	0.76 ± 0.02 ^c^
	7	0.76 ± 0.02	0.79 ± 0.01 ^ab^	0.78 ± 0.02 ^b^	0.84 ± 0.02 ^bc^
	14	0.80 ± 0.01 ^Y^	0.81 ± 0.01 ^ab,Y^	0.81 ± 0.02 ^b,Y^	0.89 ± 0.01 ^b,X^
	21	0.82 ± 0.02 ^Y^	0.87 ± 0.02 ^a,Y^	1.03 ± 0.01 ^a,X^	1.02 ± 0.02 ^a,X^
	28	0.83 ± 0.02 ^Y^	0.88 ± 0.02 ^a,Y^	1.04 ± 0.01 ^a,X^	1.10 ± 0.03 ^a,X^
**STS** **(%)**	1	42.0 ± 0.5 ^X^	37.8 ± 0.2 ^a,Y^	36.7 ± 0.3 ^a,Y^	35.5 ± 0.9 ^a,Y^
	7	42.3 ± 0.7 ^X^	39.8 ± 0.1 ^b,XY^	38.2 ± 0.5 ^ab,Y^	37.2 ± 0.5 ^ab,Y^
	14	43.6 ± 0.5 ^X^	41.2 ± 0.5 ^bc,XY^	39.3 ± 0.4 ^bc,Y^	38.3 ± 0.7 ^abc,Y^
	21	43.5 ± 0.6 ^X^	42.9 ± 0.4 ^c,X^	40.1 ± 0.4 ^bc,Y^	39.6 ± 0.5 ^bc,Y^
	28	44.1 ± 0.4 ^X^	42.6 ± 0.4 ^c,XY^	40.8 ± 0.2 ^c,Y^	41.0 ± 0.3 ^c,Y^

^a–d^ Means within a column with different lowercase superscripts in the same analysis differ significantly (*p* < 0.05). ^X–Z^ Means within a row with different uppercase superscripts differ significantly (*p* < 0.05). STS = susceptibility to syneresis; ^1^ C = yogurt produced with CH-1 culture; FLC = yogurt produced with freeze-dried free *L. casei* ATCC 393 cells and CH-1 culture; ILCA = yogurt produced with freeze-dried immobilized *L. casei* ATCC 393 cells on apple pieces and CH-1 culture; ILCC = yogurt produced with freeze-dried immobilized *L. casei* ATCC 393 cells on casein and CH-1 culture.

**Table 2:** Major aroma-related compounds (μg/kg) of novel probiotic yogurts after 14 days of refrigerated storage.

Compound	Identification Method ^1^	Yogurt ^2^			
		C	FLC	ILCA	ILCC
Esters					
Ethyl butanoate	RT, KI, MS	1.2 ± 0.2 ^a^	0.4 ± 0.1 ^b^	0.5 ± 0.1 ^ab^	ND
Ethyl hexanoate	RT, KI, MS	0.8 ± 0.2 ^a^	ND	ND	0.2 ± 0.2 ^a^
Ethyl octanoate	RT, KI, MS	ND ^3^	0.7 ± 0.2 ^a^	0.9 ± 0.3 ^a^	ND
Ethyl decanoate	RT, KI, MS	ND	ND	1.0 ± 0.2 ^a^	0.7 ± 0.3 ^a^
Organic acids					
Acetic acid	MS	25.3 ± 1.2 ^a^	31.4 ± 2.1 ^a^	52.4 ± 3.0 ^b^	51.2 ± 2.9 ^b^
Butanoic acid	KI, MS	19.2 ± 2.1 ^a^	41.4 ± 3.9 ^b^	37.7 ± 3.1 ^b^	40.5 ± 1.5 ^b^
Hexanoic acid	KI, MS	47.6 ± 3.0 ^a^	71.5 ± 4.5 ^b^	82.1 ± 5.0 ^b^	78.7 ± 3.6 ^b^
Octanoic acid	RT, KI, MS	59.2 ± 3.5 ^a^	30.9 ± 2.1 ^b^	41.5 ± 3.0 ^b^	35.4 ± 1.5 ^b^
Nonanoic acid	RT, KI, MS	9.3 ± 0.9 ^ab^	7.2 ± 0.5 ^a^	10.2 ± 0.8 ^ab^	13.4 ± 0.9 ^b^
Decanoic acid	RT, KI, MS	37.4 ± 2.2 ^a^	56.5 ± 4.2 ^ab^	65.1 ± 3.2 ^b^	70.2 ± 4.3 ^b^
2-Methyl butanoic acid	KI, MS	ND	ND	ND	4.2 ± 0.9
Alcohols					
Ethanol	RT, KI, MS	>10,000	>10,000	>10,000	>10,000
1-Hexanol	RT, KI, MS	4.2 ± 1.0 ^a^	ND	ND	3.1 ± 0.4 ^a^
1-Heptanol	KI, MS	5.1 ± 0.8 ^a^	2.1 ± 0.5 ^b^	ND	ND
1-Octanol	RT, KI, MS	ND	ND	1.2 ± 0.6 ^a^	0.7 ± 0.2 ^a^
1-Nonanol	RT, KI, MS	2.0 ± 0.5 ^a^	ND	ND	0.4 ± 0.2 ^b^
3-Methyl-1-butanol	RT, KI, MS	ND	0.7 ± 0.3 ^a^	0.4 ± 0.2 ^a^	ND
2-Heptanol	RT, KI, MS	ND	ND	1.2 ± 0.4	ND
2-Nonanol	RT, KI, MS	1.2 ± 0.6 ^c^	2.9 ± 0.5 ^bc^	5.1 ± 0.8 ^a^	4.0 ± 1.0 ^ab^
1-Octen-3-ol	RT, KI, MS	2.7 ± 0.4 ^a^	ND	1.2 ± 0.2 ^b^	ND
2-Ethyl-1-hexanol	RT, KI, MS	3.1 ± 0.3 ^a^	12.4 ± 1.0 ^b^	16.1 ± 1.2 ^b^	18.7 ± 1.6 ^b^
Phenyl ethanol	RT, KI, MS	ND	0.9 ± 0.3	ND	ND
Aldehydes					
Acetaldehyde	KI, MS	17.3 ± 1.0 ^a^	16.7 ± 1.2 ^a^	18.4 ± 0.5 ^a^	17.9 ± 1.0 ^a^
3-Methyl butanal	KI, MS	0.8 ± 0.2 ^a^	1.2 ± 0.5 ^a^	1.5 ± 0.2 ^a^	1.9 ± 0.2 ^a^
Hexanal	KI, MS	ND	2.1 ± 0.4 ^a^	ND	2.8 ± 0.3 ^a^
Heptanal	KI, MS	2.1 ± 0.4 ^a^	3.5 ± 0.3 ^ab^	4.7 ± 0.9 ^ab^	5.1 ± 0.3 ^b^
Octanal	KI, MS	5.1 ± 0.5 ^a^	12.1 ± 1.0 ^b^	12.8 ± 1.0 ^b^	14.1 ± 0.9 ^b^
Nonanal	KI, MS	9.5 ± 0.6 ^a^	10.5 ± 0.5 ^a^	14.2 ± 1.1 ^ab^	16.7 ± 1.2 ^b^
(*E*,*E*)-2,4-Heptanedial	KI, MS	ND	5.6 ± 0.4 ^a^	10.2 ± 0.8 ^b^	12.1 ± 0.8 ^b^
(*E*)-2-Nonenal	KI, MS	5.9 ± 0.4 ^a^	4.1 ± 0.1 ^ab^	7.3 ± 0.5 ^b^	6.7 ± 0.4 ^b^
Ketones					
Acetone	KI, MS	15.1 ± 0.8 ^a^	12.9 ± 1.0 ^a^	13.1 ± 1.2 ^a^	11.5 ± 0.5 ^a^
2-Butanone	KI, MS	27.3 ± 1.5 ^a^	69.4 ± 2.3 ^b^	102.1 ± 9.5 ^b^	100.9 ± 10.8^b^
2-Pentanone	MS	19.7 ± 1.0 ^a^	47.3 ± 2.0 ^b^	82.4 ± 5.5 ^c^	80.1 ± 7.0 ^c^
2-Heptanone	KI, MS	22.4 ± 2.3 ^a^	51.2 ± 1.8 ^b^	81.7 ± 4.9 ^c^	92.4 ± 6.4 ^c^
2-Octanone	MS	12.1 ± 1.1 ^a^	21.3 ± 1.2 ^b^	29.4 ± 2.2 ^b^	27.3 ± 1.2 ^b^
2-Nonanone	KI, MS	13.9 ± 1.0 ^a^	15.2 ± 1.5 ^a^	19.4 ± 1.1 ^a^	17.3 ± 0.9 ^a^
2-Undecanone	KI, MS	4.9 ± 0.5 ^a^	12.3 ± 0.8 ^b^	11.9 ± 0.8 ^b^	15.4 ± 1.0 ^b^
2-Tridecanone	MS	ND	17.4 ± 0.7 ^a^	21.5 ± 1.0 ^a^	20.4 ± 1.5 ^a^
3-Hydroxy-2-butanone(acetoin)	KI, MS	73.1 ± 5.5 ^a^	98.2 ± 7.8 ^ab^	143.2 ± 11.0 ^bc^	159.2 ± 13.8 ^c^
2,3-Butanedione(diacetyl)	MS	27.4 ± 1.5 ^a^	29.3 ± 2.2 ^a^	32.1 ± 3.0 ^a^	33.1 ± 1.2 ^a^
2,3-Pentanedione	MS	5.2 ± 2.0 ^a^	10.1 ± 1.5 ^b^	7.9 ± 1.0 ^ab^	8.7 ± 0.9 ^b^
Lactones					
γ-Dodecalactone	KI, MS	ND	1.2 ± 0.8	ND	ND
δ-Dodecalactone	KI, MS	0.5 ± 0.2 ^a^	5.1 ± 0.8 ^b^	4.9 ± 0.7 ^b^	6.2 ± 0.4 ^b^
**Total compounds**					
Esters		2.0 ± 0.4 ^a^	1.1 ± 0.3 ^a^	2.4 ± 0.6 ^a^	0.9 ± 0.5 ^a^
Organic Acids		198.0 ± 12.9 ^a^	238.9 ± 17.3 ^ab^	289.0 ± 18.1 ^ab^	293.6 ± 15.6 ^b^
Alcohols		18.3 ± 3.6	19.0 ± 2.6	25.2 ± 3.0	26.9 ± 3.4
Aldehydes		40.7 ± 3.1 ^a^	55.8 ± 4.2 ^ab^	69.1 ± 5.0 ^b^	77.3 ± 5.1 ^b^
Ketones		220.6 ± 14.7 ^a^	385.1 ± 22.3 ^ab^	544.7 ± 39.2 ^b^	566.3 ± 45.2 ^b^
Lactones		0.5 ± 0.2 ^a^	6.3 ± 1.6 ^b^	4.9 ± 0.7 ^ab^	6.2 ± 0.4 ^b^
Total		480.1 ± 34.9 ^a^	706.2 ± 48.3 ^ab^	935.3 ± 66.6 ^b^	971.2 ± 70.2 ^b^

^a–c^ Means within a row with different lowercase superscripts differ significantly (*p* < 0.05). ^1^ RT: positive identification by retention times that agree with authentic compounds generated in the laboratory; KI: tentative identification by Kováts retention index compared to the literature [14,15,26,43]; MS: tentative identification by mass spectra obtained from NIST107, NIST21, and SZTERP libraries. ^2^ C = yogurt produced with CH-1 culture; FLC = yogurt produced with freeze-dried free *L. casei* ATCC 393 cells and CH-1 culture; ILCA = yogurt produced with freeze-dried immobilized *L. casei* ATCC 393 cells on apple pieces and CH-1 culture; ILCC = yogurt produced with freeze-dried immobilized *L. casei* ATCC 393 cells on casein and CH-1 culture. ^3^ ND: not detected.
